# Photodistributed pustular acute febrile neutrophilic dermatosis revealing an acute myeloid leukemia

**DOI:** 10.1002/ccr3.5702

**Published:** 2022-04-05

**Authors:** Sarra Saad, Mouna Korbi, Nouha Ben Abdeljelil, Rim Rakez, Hichem Belhadjali, Mohamed Adnene Laatiri, Jameleddine Zili

**Affiliations:** ^1^ Department of Dermatology Fattouma Bourguiba Hospital Monastir Tunisia; ^2^ Department of Anatomopathology Fattouma Bourguiba Hospital Monastir Tunisia; ^3^ Department of Haematology Fattouma Bourguiba Hospital Monastir Tunisia

**Keywords:** acute myeloid leukemia, febrile neutrophilic dermatosis, sweet syndrome

## Abstract

Sweet syndrome is a rare inflammatory dermatosis that can be associated with various diseases, including leukemias. Physicians should be aware that a photodistributed clinical presentation of a pustular SS may reveal underlying malignancies, particularly hemopathies. If the hemopathy is known, recurrence lesions should be suspected of a relapse.

## Abstract 2

Sweet syndrome is a rare inflammatory dermatosis that can be associated with various diseases, including leukemias. Physicians should be aware that a photodistributed clinical presentation of a pustular sweet syndrome may reveal underlying malignancies, particularly hemopathies. If the hemopathy is known, recurrence lesions should be suspected of a relapse.

## 1 CASE PRESENTATION

**FIGURE 1:**
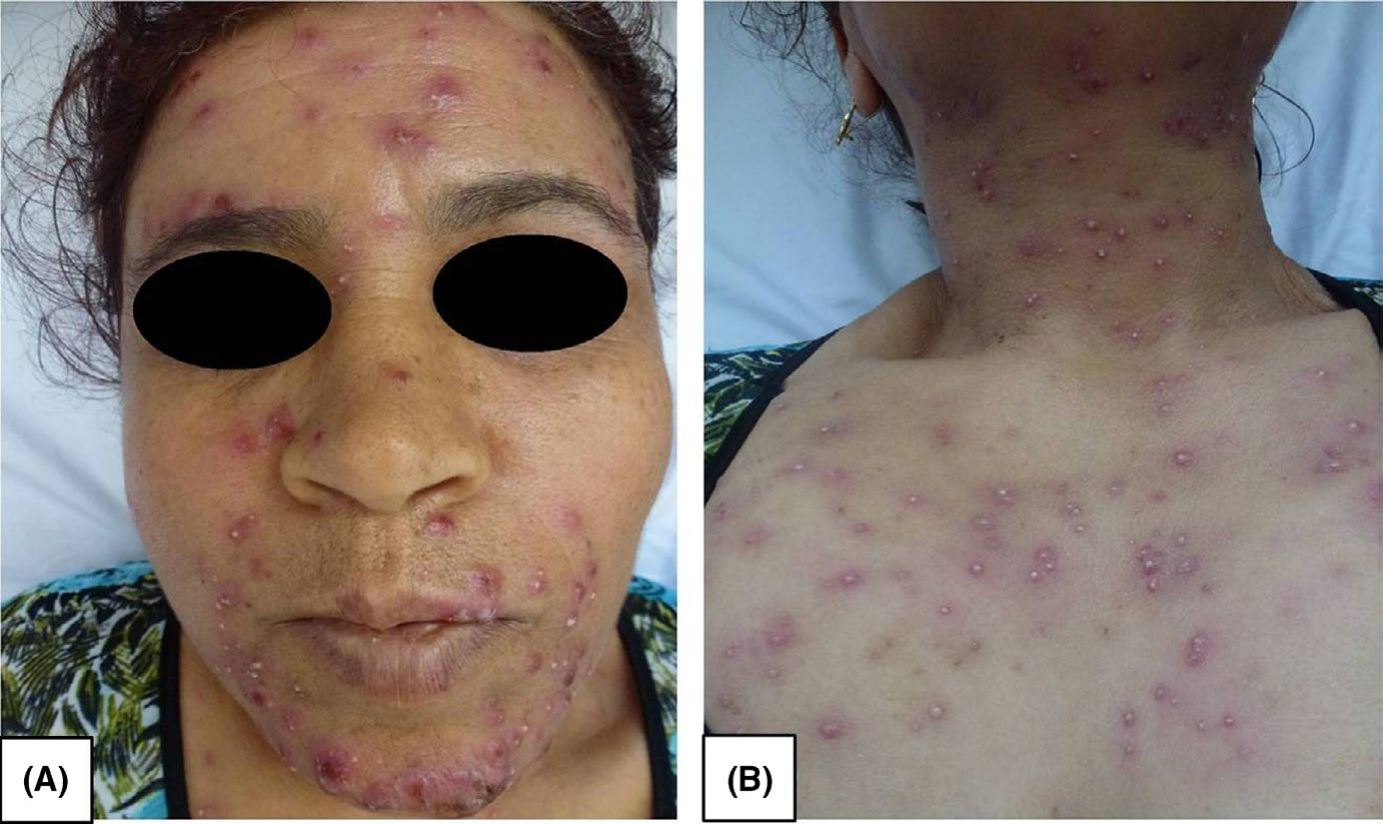
(A) Erythematous and pustulosis lesions on the face. (B) Erythematous and pustulosis lesions on the chest with a sharp cut‐off between the lesions and the photoprotected area

**FIGURE 2:**
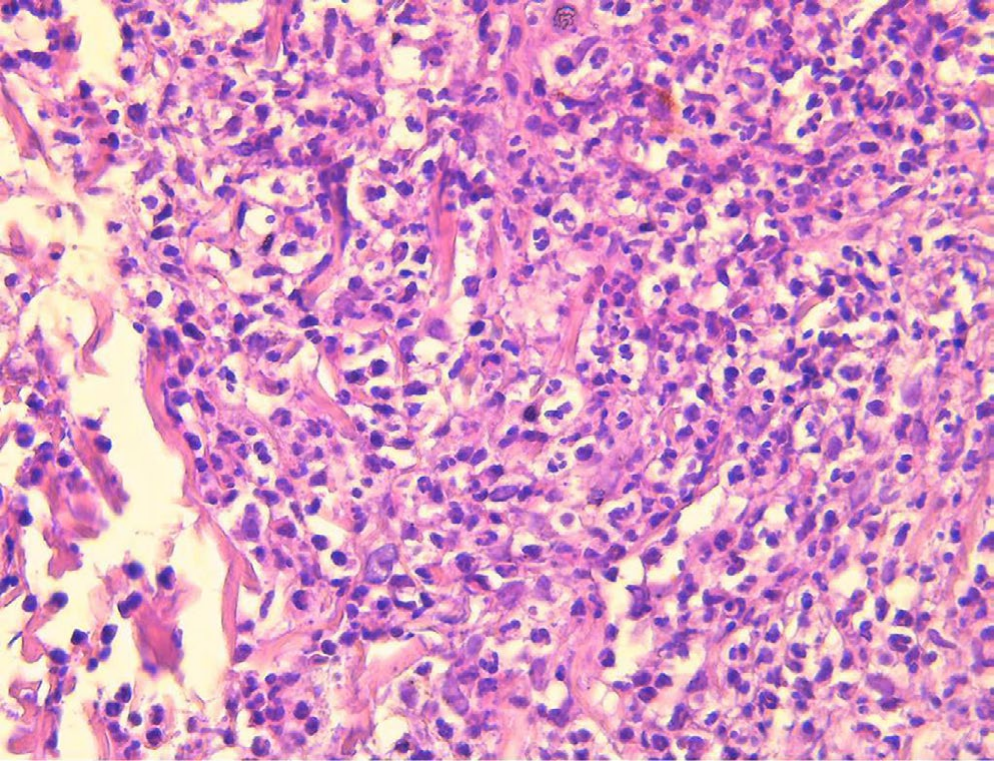
Intense neutrophilic dermal infiltrate with marked leukocytoclasia (HE×400)

A 52‐year‐old woman, presented with a 5‐day history of painful erythematous and pustulosis lesions on the face and the chest, associated with fever and arthralgia (Figure 1A). The lesions were well limited in the photoexposed area (Figure 1B). The histological examination was in favor of the diagnosis of sweet syndrome (SS) (Figure 2). Biological investigations revealed white blood cell count 22.73 10^3^/μl with predominantly neutrophil and blasts of myeloid appearance with a cytoplasm showing azurophilic granulations and sometimes Auer bodies. The myelogram confirmed the diagnosis of acute myeloid leukemia (AML). The patient was treated with chemotherapy and dexamethasone resulting in the complete healing of cutaneous lesions within a few weeks.

## 2 DISCUSSION

Sweet syndrome (SS) is characterized by painful cutaneous nodules and neutrophilic infiltrate in the dermis without vasculitis.^1^, ^2^ SS in photoexposed regions, as reported in our patient, has been described in few patients diagnosed with drug‐induced SS and idiopathic SS.^1^

The atypical clinical presentation of SS is usually associated with paraneoplastic origin. Although the neutrophil infiltrates are morphologically mature, their original association with leukemia remains undefined.^2^

Physicians should keep a high index of suspicion in front of a pustular photodistributed SS and search for underlying malignancies especially hemopathies, or relapse if the tumor is known.

## CONFLICT OF INTEREST

None.

## AUTHOR CONTRIBUTIONS

Dr Saad wrote the first draft of the manuscript and took clinical pictures. Dr Korbi and Dr Belhadjali helped in writing the manuscript and literature search. Dr Ben abdeljelil contributed to the histological data. Dr Rakez and Dr Laatiri contributed to the hematological data. Dr Zili revised and approved the final version of the manuscript. All the authors contributed to and have approved the final manuscript.

## ETHICAL APPROVAL

None.

## CONSENT

Written informed consent was obtained from the patient to publish this report in accordance with the journal's patient consent policy.
